# How Free Is Free Health Care? An Assessment of Universal Health Coverage Among Jamaicans with Sickle Cell Disease

**DOI:** 10.1089/heq.2021.0002

**Published:** 2021-04-21

**Authors:** Zachary J.A. Ramsay, Rachel E. Bartlett, Christine A. Clarke, Monika R. Asnani, Jennifer M. Knight-Madden, Georgiana M. Gordon-Strachan

**Affiliations:** ^1^ Caribbean Institute for Health Research—Sickle Cell Unit, The University of the West Indies Mona Campus, Kingston, Jamaica.; ^2^ The Department of Economics, The University of the West Indies Mona Campus, Kingston, Jamaica.

**Keywords:** out-of-pocket payment, universal health coverage, sickle cell disease, universal health care, health financing

## Abstract

**Purpose:** In an effort to transition toward universal health coverage (UHC), Jamaica abolished user fees at all public health facilities in 2008. We aimed to determine the extent of out-of-pocket payments (OPPs) and the other cost barriers to UHC among patients with sickle cell disease (SCD).

**Methods:** Patients presenting to the Sickle Cell Unit in Kingston, Jamaica, for routine care between October 2019 and August 2020 were consecutively recruited and interviewed about their latest hospitalization within the previous 4 weeks. Parents or guardians completed the questionnaire on behalf of pediatric patients. The questionnaire included the Patient Satisfaction Questionnaire Short Form (PSQ)-18 and the health module of the Jamaica Survey of Living Conditions.

**Results:** There were 103 patients with ages ranging from 7 months to 56 years (51.5% female, 60.2% public hospitalizations, and 54.4% pediatric). The modal income (J$6200–$11,999 per week) was similar to the minimum wage and 48.5% lived in overcrowded households. Government drug-subsidy cards were owned by 39.8%. OPPs were made by 19.4% of persons for items and tests that were unavailable at public facilities. There were no costs reported by 69.6%, who visited public pharmacies. Similarly, the cost of admission to public hospitals was free for 95.4% of subjects. Using public transportation, private hospitalization, and having more disease complications were predictive of a perception that health care is unaffordable.

**Conclusion:** Most SCD subjects reported no expense with public hospitalizations; however, approximately one in five reported OPPs. Efforts are needed to increase the availability of subsidized items, and the use of drug-subsidy cards, to improve UHC.

## Introduction

Universal health coverage (UHC) is one of the targets of sustainable development goal three and aims to ensure that “all individuals and communities receive the health services they need without suffering financial hardship.”^1^ Jamaica's UHC was last estimated in 2017 at 65%, which is below the approximate mean of 75.4% in Latin America and the Caribbean.^2,3^

Jamaica abolished user fees in the public health sector for all persons in April 2008. The National Health Services Act 1997 (amended in 2016) provides exemptions of fees for patients using public health facilities, who are resident in Jamaica for all treatment modalities, including surgery, imaging and laboratory investigations, and pharmacy services, however, with the exception of medical reports, purified protein derivative tests, immunization for international travel, full medical screens, and morgue services.^4^ Patients who are residents in Jamaica also have the option of paying the fees.

There have been varying opinions and findings of the policy. Garzon and Beuermann^5^ compared the Jamaica Labour Force Survey and the Jamaica Survey of Living Conditions (JSLC) between 2002 and 2012 and found economic benefits, including an estimated 2.15 h added to the labor week and US$ 26.6 million purchasing power parity of net real production added to the economy in 2008–2012. The Medical Association of Jamaica reported many practical problems, particularly poor working conditions and low resources, and recommended continuing the no-user fee policy only for children, elderly, and the mentally challenged.^6^

Out-of-pocket payments (OPPs) are defined by the World Health Organization (WHO) as “direct payments made by individuals to health care providers at the time of service use,” with the exception of prepayments such as health insurance or taxes.^7^ It is an important indicator of health expenditure, especially in the developing world where it contributes 45% of the total health expenditures and may force persons into poverty.^8^ JSLC is a nationally representative survey of living standards, which is fielded annually. It monitors health care expenses of the previous 4 weeks and its last estimate of the proportion of OPPs among those visiting public facilities in 2014 was found to be 2.4%, with the average nominal cost being J$1727 (US$ 15.52).^9^ The last published survey in 2017 found the mean cost of medication at public pharmacies to be J$162 (US$ 1.26).^10^

The health system in Jamaica comprises public facilities that are free at the point of service, and private facilities that charge a user fee. The Sickle Cell Unit (SCU) is an outpatient clinic that provides emergency and routine clinic care for sickle cell disease (SCD) patients from all sections of the island. The University Hospital of the West Indies (UHWI) and the SCU charge user fees and are both considered private institutions. Prescriptions and requests for diagnostic tests written at the SCU or UHWI cannot be filled at government-subsidized pharmacies and other public facilities. SCD patients often have difficulty accessing specialist care because the SCU is often distant from home and there is a paucity of specialty clinics within local hospitals.^11^ The Bustamante Children's Hospital (BCH) and the Kingston Public Hospital (KPH) are the closest public hospitals to the SCU for pediatric and adult patients, respectively. The National Health Fund (NHF) drug-subsidy cards cover the full cost of medication that is on a subsidized list in public pharmacies, and part of the cost in some private pharmacies.

SCD is a common hemoglobinopathy in Jamaica, which occurs in 1 in 150 live births.^12^ The disease is characterized by many complications resulting in high health care utilization^13^ and worse health care disparities.^14,15^ Therefore, the burden of the disease is significant, and this presents an important population to study.

There remains evidence of OPPs in Jamaica, and this may present as a barrier to UHC among SCD patients who are already disadvantaged. We conducted this study to determine the OPPs made by SCD patients, while admitted to public hospitals in Jamaica. The achievement of UHC involves more than just financial protection and affordability, and other dimensions, including quality, access to all services, and facilitating access for persons with differential circumstances.^16^ We therefore also aimed to assess these dimensions and determine the demographic, socioeconomic, and clinical factors that may present as barriers to UHC. These factors included patient satisfaction, delays in accessing health care, and transportation. Furthermore, we intended to compare the health care costs and level of satisfaction among SCD patients admitted to public versus private hospitals in Jamaica. Significant differences identified at public hospitals may represent barriers to UHC.

## Methods

A cross-sectional study was conducted at the SCU between October 2019 and August 2020. The study was approved by the University of the West Indies Mona Campus Research Ethics Committee (ECP 113, 18/19).

Subjects were consecutively recruited if they had no acute illnesses at the time of the visit and were hospitalized in the previous 4 weeks. Each participant had data collected for only their last hospitalization. After informed consent, subjects completed an interviewer-assisted questionnaire in person or by telephone.

The study instrument that ascertained general health expenditure and usage was a modified version of the health module used in the JSLC.^10,17,18^ The JSLC has been validated against the Survey of Older Men and California Achievement Test.^19,20^ Data collected included the name of the hospital, diagnosis, and any OPP made associated with the hospitalization. We assessed delays in accessing care as the number of days between becoming ill and visiting hospital for hospitalization. Subjects also completed the Patient Satisfaction Questionnaire Short form (PSQ), which assesses patient's level of satisfaction with health care received during the hospitalization based on 18 items, each gauging domains of health care experiences using Likert-scale responses. Higher scores on the PSQ indicate greater satisfaction, with a possible range of 0 to 90. The PSQ was originally validated against the long-form PSQ-III, with internal reliability coefficients and correlations all greater than 0.6.^21^ The long-form PSQ has been validated among Jamaicans with SCD, with good internal reliability coefficients of 0.70 or more.^22^

### Classifications of SCD and its severity

Genotypes were defined as mild when SC or Sβ-thalassemia^+^ and severe when SS or Sβ-thalassemia^0^. SCD complications were assessed using the disease severity checklist module of the Adult Sickle Cell Quality of Life Measurement Information System, which has been validated against the Patient-Reported Outcomes Measurement Information System with median correlations of 0.69.^23^ The checklist assessed the presence of nine important SCD complications, and we used a score based on the sum of all the domains (possible range: 0–9).

Expenditures reported were direct costs associated with care, such as diagnostic tests, medical equipment, and medication. Subjects were instructed not to include indirect costs such as transportation and food when reporting actual expenses, although these may be reflected in their subjective reporting of health care affordability. Parents or guardians completed the questionnaire on behalf of pediatric patients (less than 18 years old), in which case the reports of costs and ability to pay were related to the parents or guardians who provide for them.

### Statistical analysis

A crowding index was calculated as the ratio of the total persons to the total number of habitable rooms (excluding kitchen and bathroom) in the household; and households were “overcrowded” based on the definition of the JSLC of the crowding index greater than 1.1. Means with standard deviations and medians with interquartile ranges (IQRs) were compared using Pearson's chi-squared tests, *t*-tests, analysis of variance (ANOVA) regressions, Wilcoxon rank-sum, Spearman's rank-order correlation, simple logistic regression, and Kruskal–Wallis tests, where appropriate.

Unaffordability of hospital expenses was categorized into “Agree,” “Disagree,” and “Uncertain” from responses to item 7 of the PSQ. Multinomial logistic regression and Pearson's chi-squared test were used to assess this main outcome in bivariate analyses. In multivariate analyses, likelihood ratio tests and improvements in log-likelihood were used to assess initial models, which were built in a backward stepwise direction beginning with variables that had *p*-values of <0.25 on bivariate analyses or were clinically significant. All analyses were done using Stata 14.2.

## Results

We assessed 103 subjects (51.5% female; 45.6% adult) with mean age of 20.2±17.8 years (range: 7 months–56 years), and with 62 (60.2%) being public and 41 (39.8%) being private hospitalizations. Socioeconomic sample characteristics are summarized in Table 1.

**Table 1.** Socioeconomic Characteristics of Sickle Cell Disease Patients Hospitalized in the Last 4 Weeks

Socioeconomic characteristics	Frequency (%)
Community	
Urban	77 (74.8)
Rural	26 (25.2)
Employment of adult patients	
Employed	27 (57.5)
Unemployed and not looking	12 (25.5)
Student	5 (10.6)
Unemployed and looking	3 (6.4)
Highest level of school completed by adult patients	
Primary or less	8 (17.0)
Secondary or technical/vocational	24 (51.1)
Tertiary	15 (31.9)
Transportation method	
Public	69 (67.0)
Private	34 (33.0)

Overcrowded homes were reported by 48.5% of the sample. Weekly income earned by all adults in the household is shown in Table 2, and there was significant nonreporting of income.

**Table 2.** Weekly Income of All Adults in Household and Respective Crowding Index Reported by Jamaicans with Sickle Cell Disease

Household income per week ($J)	Frequency (%)	Median crowd index (IQR)
More than 60,000	4 (3.9)	0.9 (0.4–1.5)
23,001–60,000	13 (12.6)	1 (0.6–1.2)
12,000–23,000	15 (14.6)	1.5 (1–2)
6200–11,999	18 (17.5)	1.1 (0.75–1.8)
Less than 6200	14 (13.6)	1.2 (0.7–2)
Do not know or no response	39 (37.9)	1 (0.8–1.7)

Exchange rate: the annual average exchange rate for US$ 1.00 was J$ 134.22 in 2019.

IQR, interquartile range.

### Clinical descriptions

Most persons were of a severe genotype (*n*=91, 88.4%) compared to mild (*n*=12, 11.7%), including SS (87.4%), SC (6.8%), Sβ-thalassemia^+^ (3.9%), and Sβ-thalassemia^0^ (1.0%). Hydroxyurea was being used by 23 (22.3%) persons. Approximately 52.4% had one or more disease complication (median: 1; IQR: 0–2; range: 0–7), including leg ulcers (21.4%), regular blood transfusions (15.5%), kidney damage (13.6%), spleen removed or damaged (11.7%), retinopathy (11.7%), daily pain medication (9.7%), lung disease (8.7%), shoulder or hip damage (7.8%), and stroke (6.8%).

These admissions were reported from these hospitals: UHWI (41, 39.8%), BCH (37, 35.9%), KPH (7, 6.8%), and smaller district hospitals (18, 17.5%). The mean duration of hospitalizations was 11.2±11.9 days (range: 1–58). The major reasons for hospitalizations, of which individuals sometimes reported more than one, were acute chest syndrome (37.9%), severe painful crisis (24.3%), acute anemia (15.5%), hepatic sequestration (12.6%), kidney failure (4.9%), and splenic sequestration (4.9%). Three persons had surgery during their hospitalization and another three were admitted to the intensive care unit—who were all admitted to public hospitals and reported no costs. Public hospitals (40.2%) and the SCU (29.4%) were the most common location that patients reported they usually first sought care when sick as opposed to private doctors/hospitals (23.5%) or public health centers (6.9%).

### Health care costs

Private pharmacies were more commonly utilized (55.9%) compared to public (44.1%). Most persons (69.6%) who visited public pharmacies reported no costs; however, 6 persons reported spending less than J$ 7500. When the cost of medications was excluded, most persons had no direct expenses when admitted to public hospitals (95.4%).

**FIG. 1.**
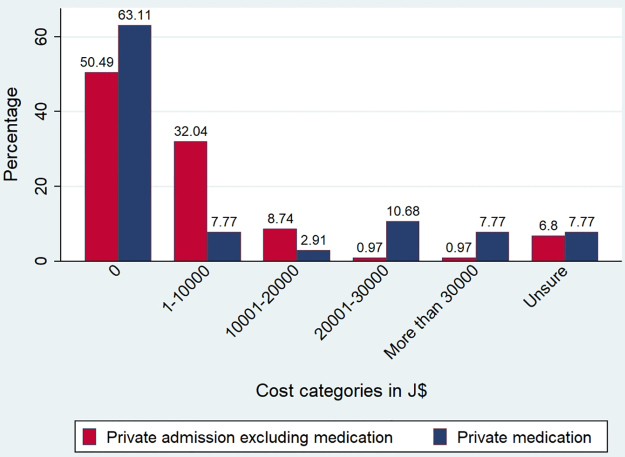
Private health care costs reported by Jamaicans with SCD over a 4-week recall period. Private pharmacy costs reported in the last 4 weeks may have been reported by persons admitted to both public and private hospitals. Exchange rate: the annual average exchange rate for US$ 1.00 was J$ 134.22 in 2019. SCD, sickle cell disease.

Private health care costs, including hospitalizations and medications, were reported as shown in Figure 1. Most persons reported spending at the SCU in the previous 4 weeks (76, 73.8%), including the J$500 registration fee to be seen at one visit at the SCU and the cost of some vaccines (mean: J$646.1±474.5, range: J$500–3500).

There were 16.5% of persons with health insurance and 39.8% owned NHF drug-subsidy cards. Of those with an NHF card, 26.8% used it with this hospitalization, and 81.8% reported that it provided only minimal savings or none at all (as opposed to “moderate or significant,” 18.2%).

There were 14 persons who purchased items, while admitted to public hospitals. OPPs were made by 19.4% of persons admitted to public hospitals for items and tests that were unavailable at the facility. The specific items listed when unavailable were antibiotics cardiovascular medication, including carvedilol, hydrochlorothiazide, and ivabradine; diagnostic tests, including dengue virus antibody tests, X-rays, and blood cultures; SCD-specific medication, including hydroxyurea and folic acid; opioid analgesics, including oral meperidine and paracetamol/codeine; and medical equipment: wound dressing and incentive spirometer.

Among the items that were unavailable at the facility, about half of these were to be used during hospitalization (9, 52.9%) as opposed to items or tests needed after discharge from hospital (8, 47.1%). Also, four subjects (23.5%) reported that they paid less than $J 2500.00 for OPP. BCH (29.4%) was the most common hospital where persons were admitted and had OPPs for items or services that were unavailable.

### Patient satisfaction

**FIG. 2.**
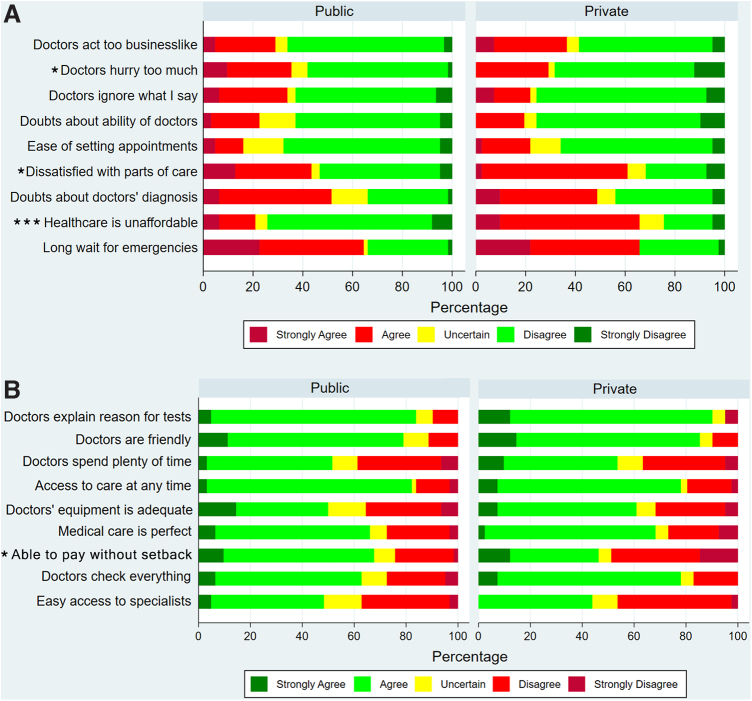
Satisfaction scores for patients admitted to public and private hospitals with SCD in Jamaica. Each PSQ item listed has been paraphrased. (**A**) Negative Satisfaction Domains. (**B**) Positive Satisfaction Domains. Pearson's chi-squared tests: *p<0.05; ***p<0.001. PSQ, Patient Satisfaction Questionnaire Short form.

The overall mean satisfaction score was 58.5±9.2, with no difference between public and private hospitalizations (58.8 vs. 58.1, *p*=0.731). Please see Figure 2.

### Disease severity

There was a positive correlation between disease severity and private health expenses (Table 3). The odds of having OPPs during a public admission were not significantly associated with disease severity (odds ratio [OR]=−0.1, confidence interval [CI]: −0.5 to 0.4; *p*=0.68).

**Table 3.** Correlations of Private Health Care Expenses with Disease Severity

Cost categories	Disease severity score: median (IQR)
Private admission costs	
0	0 (0–1)
1–10,000	1.5 (0.5–3.5)
10,001–20,000	0 (0–1)
20,001–30,000	2 (1–3)
More than 30,000	2 (0.5–3)
Unsure	1 (0.5–2)
*r*_s_, *p*	0.35, <0.001
Private medication costs	
0	0 (0–1)
1–10,000	1 (0–2)
10,001–20,000	1 (0–3)
More than 20,000	3 (2–4)
Unsure	1 (0–2)
*r*_s_, *p*	0.27, 0.006

Statistical test: Spearman's rank-order correlation.

“Unsure” describes subjects who could not recall the expenses or those who had outstanding bills.

### Public versus private hospitalizations

There was no association between the type of hospitalization, whether public or private, with either employment status (*p*=0.91) or the highest level of education achieved (*p*=0.37) among adults. Overall, the reported numbers of visits to any health care facility in the previous 12 months were less than 6 (22.3%), between 6 and 11 (35.9%), 12 or more (22.3%), and unsure (19.4%), and there was no difference between persons who admitted to public or private hospitals (*p*=0.66). Persons visiting for public hospitalizations were likely to be in the pediatric age group (*p*<0.001) and live in overcrowded homes (*p*=0.005). The odds for being admitted to a private hospital were higher when persons had more disease complications (OR=1.9; CI: 1.3 to 2.7; *p*=0.001). There were no other significant public and private differences for sex (*p*=0.66), hydroxyurea use (*p*=0.37), genotype (*p*=0.63), community type (*p*=0.87), or means of transportation (*p*=0.05).

### Health care affordability

As many as 38.8% of subjects reported a perception of being unable to afford health care costs associated with the last admission, and this was significantly associated with private hospital hospitalizations as opposed to public (*p*<0.001) and more disease complications (OR=1.9; *p*<0.001, CI: 1.3 to 2.7). There were no associations with hospitalization diagnoses (all *p*>0.05); sex (*p*=0.54), community type (*p*=0.41), presence of overcrowded home (*p*=0.09), type of transportation (*p*=0.08), genotype (*p*=0.94), or hydroxyurea (*p*=0.76). Among adults, there was also no association with employment status (*p*=0.95) or the highest level of education achieved (*p*=0.41).

### Delays in accessing care

There was a median delay in accessing health care before the hospitalization of 1.5 days (IQR: 1–3). There was no association between the delay in days to seek care with a perception of health care affordability (*p*=0.31), public or private hospitalizations (*p*=0.62), or the type of transportation (*p*=0.62).

### Predictors of reporting health care affordability

The adjusted odds of a perception of health care being unaffordable with the last admission were higher when the hospitalization was private, when subjects used public transportation, and when there were more disease complications (Table 4).

**Table 4.** Multinomial Logistic Regression of Reports of Ability to Pay for Medical Care by Sickle Cell Disease Patients Hospitalized in Jamaica Between October 2019 and August 2020

Outcomes	Predictors	Odds-ratio (SE)	Confidence interval
Perception that health care was unaffordable at last admission (40, 38.8%)	Private hospitalization (reference: public hospitalization)^***^	8.0 (4.3)	2.8 to 22.9
	Nonprivate transport (reference: private car)^*^	4.2 (2.6)	1.3 to 13.8
	Number of disease complications^*^	1.6 (0.3)	1.0 to 2.3
Uncertain about health care affordability (7, 6.8%)	Private hospitalization (reference: public hospitalization)^*^	8.0 (7.1)	1.4 to 45.6
	Nonprivate transport (reference: private car)	1.0 (0.8)	0.2 to 5.2
	Number of disease complications	0.7 (0.3)	0.3 to 1.7

Pseudo *R*^2^=0.219 LR chi-squared=39.7 (*p*<0.001); reference outcome: perception that health care was affordable at last admission (*n*=56); ^*^p<0.05, ^***^p<0.001.

SE, standard error.

A sensitivity analysis using logistic regression modeling was done to compare the results if the responses of “uncertain” were excluded, and there were no differences in which predictors were statistically significant.

## Discussion

We aimed to determine the proportion of SCD subjects who make OPPs, while admitted to public hospitals in Jamaica, and found OPPs were made by 19.4% of these persons for items and tests that were unavailable at the facility. In Nigeria, as many as 74.9% of SCD health expenses were paid as OPPs,^24^ compared to only 5% in the United States.^25^ These country-specific differences are likely due to the complex differences in health financing. Unfortunately, only 39.8% of subjects owned NHF cards, which is comparatively worse than the eligible general population where about 50% owns an NHF card.^10^ More efforts are needed to increase the uptake and usage of NHF cards by patients at the SCU. Considerations also need to be made to ensure that common medications and tests, which were frequently needed, are kept in stock at government facilities.

Other barriers to UHC, separate from OPP, were also examined. Most importantly, SCD patients were found to have relatively low socioeconomic status. We found that more SCD families live in overcrowded homes than the general Jamaican population (48.5% vs. 37.4%).^10^ Most persons, who were able to recall an amount, reported that all adults in the household made between J$6200 and $11,999, which is closely aligned with the national minimum wage of J$7000 per week. SCD patients having relatively low socioeconomic status is a consistent finding in the literature. In Nigeria, 32.9% of the families with SCD children studied survived below the poverty threshold and 63% made only minimum wage.^24^ As many as 68.8% of SCD subjects in a study in Brazil were unemployed, with as many as 87.5% living below the poverty line.^26^ In keeping with lower socioeconomic status, more SCD subjects in our study report being unable to afford health care (38.8%), compared to 12.2% of the general population surveyed in the JSLC in 2017.^10^ Many factors may account for this, particularly higher health care utilization among SCD subjects for acute and chronic complications. Similarly, Medicaid expenditure in the United States has been found to be more than eight times higher for SCD children in comparison to all children on average.^27^

We also found that using public transportation was predictive of reports of health care unaffordability. Using public transportation is likely a proxy for low socioeconomic status; however, the impact of transportation access and costs may be significant. In Jamaica, Gordon-Strachan et al.^28^ have reported that lack of access to transportation was a barrier to accessing primary care for elderly persons with chronic diseases. Among Nigerian pediatric SCD patients, transportation costs were found to be as much as 9.8% of all annual costs associated with health care.^24^ Subjects with more disease complications also reported health care being unaffordable, which is not surprising as these patients are expected to have more health care visits and medication to pay for. Similarly, among 10,784 subjects in the United States, SCD patients with end-organ damage had “higher all-cause health care costs and more inpatient days, emergency department visits, outpatient visits, laboratory tests, and outpatient pharmacy claims” than those without; and costs were higher for those with multiple end-organ damage.^29^

We also aimed to compare subjects who were admitted to public and private hospitals. Persons from overcrowded households, a proxy for low socioeconomic status, were more likely to be admitted to public hospitals. This suggests that the expensive costs of private health care may impact health care decisions and preferences, but requires further assessment. More studies are also needed to understand the preference for public hospitals among pediatric patients and those with more disease complications.

The major limitation of this study is that we did not have access to bills, and we had to rely on patients' reports of expenses, and many were unable to recall. Costs were therefore categorized, and we focused on subjective reports of health care affordability as our primary outcome.

## Conclusion

Most SCD subjects reported no expense with public hospitalizations; however, approximately one in five reported OPPs. Efforts are needed to increase the availability of subsidized items, and the use of drug-subsidy cards, to improve UHC.

## Abbreviations Used

ANOVA: analysis of variance
BCH: Bustamante Children's Hospital
CI: confidence interval
IQR: interquartile range
JSLC: Jamaica Survey of Living Conditions
KPH: Kingston Public Hospital
NHF: National Health Fund
OPPs: out-of-pocket payments
OR: odds ratio
PSQ: Patient Satisfaction Questionnaire Short-form
SE: standard error
SCD: sickle cell disease
SCU: Sickle Cell Unit
UHC: universal health coverage
UHWI: University Hospital of the West Indies
WHO: World Health Organization
